# Trichinæ in American Pork

**Published:** 1884-07

**Authors:** 


BILLINGS.

### TRICHINÆ IN AMERICAN PORK.

Our attention is again called to this subject by the Report of the Commission on the Swine Products of the United States lately nominated by the President. The "Report" is an able one; it is not going too far in saying that it is the ablest and most satisfactory one that we have yet seen emanating from our Government on any subject in connection with the disease of our domestic animals. It should be obtained and read by every veterinarian in the country. While it contains much valuable information, and thoroughly proves that the assertions of some European authorities "that the refuse from our large slaughter and packing-houses is either fed to hogs that are kept at such places, or sold to outside hog-feeders," is absolutely false, still it is very meagre upon the question of all questions which this Commission was especially appointed to investigate. We allude to the Trichiniasis question. It must be borne in mind that we look upon this question far more from an American than a European standpoint; though we have seen fit, unpatriotically, it is asserted, to defend the action of foreign governments in shutting out American pork on account of trichiniasis.

We make bold to assert that so far as our personal examinations have extended, American pork is more highly infected with trichinæ than that of any other country where examinations have been made.

Further: There are as natural reasons why this must, or should, be the case, as why the German people are more prone to trichiniasis than any other nation. The Germans will eat raw, highly spiced hashed pork; they also eat, as a delicacy, finely sliced, raw smoked ham, as we do chip beef. They suffer the penalty, notwithstanding the great number of pork examiners which they have. The American and Englishman will eat raw, warm beef; if he has *tænia saginnata* (a tapeworm) for his "cannibalism," it is his own fault. But this is begging the question.

We have said that there are natural American causes why our pork should be more trichinous than most European or English. This has wholly escaped the attention of our Commission. In fact they take a contrary view, and look upon it as favoring the healthy condition of the American hog. In many ways we agree with them; but having given as much, if not more, study to Trichiniasis than any man in the country, we are forced to the conclusion that it is the cause of American pork being so highly trichinous. They say rightly that "the disease of trichiniasis is practically unknown among the farmers; and we fail to see how corn, the chief food for hogs which make up the market supply, can in any possible way be productive of trichinosis." How should the farmers know this disease when even the doctors often fail to recognize it in the human being? The microscopic examination of the flesh is necessary to recognize its presence, and some technical ability besides. Any hog could have a diarrhœa, followed by lameness or rheumatism, and recover; for hogs generally do. The farmer would almost naturally attribute such a thing to a cold.

But the key to the trouble must be sought here. The Report says: "Where the climate is milder the hogs are sometimes kept in the forests." Again: "The hogs in the United States are generally allowed to roam, and feed upon grass and clover as their natural food, during those months in which pasturage can be continued"; "the pigs of this kind are farrowed in the summer while the mothers are in the fields or woods; when in the fields they live, as we have said, upon blue grass and clover; in the woods or on the river bottoms they subsist upon grass and roots of various kinds."

Here is the "root" of the whole matter. We think, but do not know, that it is this very grazing that causes so much trichiniasis in American pork. The hog is notedly omnivorous; "roots, nuts, and grasses" are not the only things that it finds to eat while seeking food in the woods and marshes. Grubs, worms, and reptiles are eaten with equal gusto. Grain never yet caused trichiniasis. Who among the many dog owners of the country would think that the dog infests itself, by gnawing its own skin, with the necklace tapeworm (*tænia cucumerina*)? Yet such is the case. Who of them would seek the cause of this parasite in a microscopically small embryo that lives within the body of a small louse that infests the dog's skin, and by this gnawing for itching gets into the dog's mouth, and from thence into the intestines, where the digestive processes set the embryo free, and it develops into a large and troublesome tapeworm?

It is a similar case with which we have to deal in trichiniasis of our hogs. Trichinæ do not develop from free eggs attached to the feed of the hogs. The embryo must live in some other living object. This object is taken up in its rooting by the hog. It becomes free in the same manner as the tapeworm embryo above described ; but here the similarity ends. The trichin-embryo does not go on developing, and itself invade the pig's muscles. It is an embryo ; it matures within two days ; sexual connection between males and females next occurs ; in from five to seven days the female produces from 1,500 to 2,500 living young embryos ; the parents die, and in general cause no more harm ; it is the embryos that cause trichiniasis ; they pass through the walls of the intestines into the adjoining muscles, and from thence into the other muscles of the body; they are still embryos ; they remain so ; they become imprisoned in the fibre of the muscle ; they are inert ; they cause no further harm to this organism, and may remain in this inert condition until the hog dies, or is killed. But if man, a dog, or some other warm-blooded animal eats of this pork, the same completion of development, birth of young, and invasion of man's organism occurs as has been related of this parasite in the hog.

What leads us to the conclusion that the cause of such a percentage of trichiniasis in American hogs is to be sought in our grazing system, is the fact that the parasite has been so frequently found to infest wild hogs that subsist in this way only. That such has occurred is very strongly emphasized by the experience of Dr. Wortabet on the Jordan, in Palestine, where a whole village partook of the flesh of a wild boar, and not a single person that eat its meat escaped trichiniasis. Many of them died.

That American hogs are more trichinous than those of Germany, is proven by the Report in question. "Taking all the examinations of American pork thus far made, both at home and abroad, we have a total of 298,782, trichinæ being found 6,280 times, or 2.1 per cent., or 1 out of every 48."

The Commissioners do not, in the above, do justice to the American hog, for the German examinations of our pork have no statistical value whatever, being made on pieces, hams, shoulders, and sides, several of which may have belonged to the same hog. These examinations do, however, have a certain value when similar ones are made upon like pieces of German hogs, and they show greatly to the disadvantage of American pork. In my own examinations of Western hogs, made in Boston, 8,773 being examined, I found 1 out of every 25

trichinous, or 4 per cent. From 1876 to 1881, 12,816,831 hogs were examined in Prussia for trichinæ, 6,945 being trichinous, or 1 out of every 1,845.

We must accept these figures notwithstanding the numerous outbreaks of trichiniasis in human beings in Germany, even when the pork has been reported to have been examined. While I believe that an average of at least one out of every hundred of our Western hogs will surely be found trichinous, yet I do not think the same figures will hold good for Eastern, or hogs that are never grazed. I do not wish to make any excuse for the American hog, any more than I desire to create a prejudice against Western pork.

A fact exists ; investigations have only been made on Eastern pork. The logical result can be nothing else than that it must suffer in consequence, until either our National or State authorities organize a rigid inspection of at least 10,000 hogs in every State. For my part, in the interests of public health and common sense, I should like to see the case proved against the filthily housed and fed hog of the small farmer and householder of the East, rather than what should be the much cleaner reared hog of the West. But it will not be so, and the grazing business is, to my mind, the reason why it will not be. We have frequently seen it asserted " that not a single case of trichiniasis in human beings in Germany could be directly traced to the consumption of American pork."

This is begging the question again. So much the better if it is so. It does go to prove that our curing processes are well done, but proves nothing as to the prevalence of trichinæ in our pork. Have our Governments no responsibilities towards our own people? Is not the duty of our own representing authorities, whom we elect for just such purposes, to protect us from eating dangerously infested or infected food? Have we had no cases of trichiniasis among our own people?

In my book "On the Relation of Animal Disease to the Public Health," I have collected the following cases :

Dr. Kiefer of Detroit, a fatal case.

Dr. Herr of Dubuque, Iowa, 15 cases ; 5 deaths.

Several cases in the "American Journal of Medical Science."

January, 1881, a case at Blackwell's Island.

Dr. Germer, Erie, Penn., 7 cases.

Dr. Sutton, Aurora, Ind., 9 cases ; 3 deaths.

Brooklyn, N. Y., 7 cases ; 2 deaths.

"Rochester Democrat," May, 1, 1879, several cases.

Annals of Michigan Board of Health, several cases.

Three cases lately occurred at Brockton, Mass., and others have occurred at Framingham, Lowell, etc., in the same State.

These are but a few cases collected in casual reading; but since public attention has been called to the matter, we find them much more frequently recorded in the daily journals than formerly. Whether they be more or less, they more than answer our purpose, which is to show that our State and local authorities should be obliged, by public sentiment, to make and execute laws forbidding the cutting up or sale of hog meat before it has been pronounced non-trichinous by an appointed inspector, the competency of whom had been at first assured by the local Board of Health.

"Cooking kills them," is the next excuse. But they may not all be "cooked," and a small number can produce thousands, with the resulting disease. No one desires to eat worms, much less when a dangerous disease can follow such an act. Our Government has treated this question all along from the wrong standpoint. It has acted as if the German citizen was the only consumer of American pork, and as if it would drive it down his throat whether he would have it or not. The "Ochiltrees" of our Congress have talked about the "protective endeavors" and agrarian opposition of Germany. Pretty arguments from a Government that is the ultra-protectionist of the world! We had better begin at home, and seek to protect our own people from eating trichinous pork before we find fault with Germany for truly trying to do for its people what we should for our own.

That American pork is trichinous is admitted by this very Report. The cause is to be sought in the surroundings and natural, not artificial, feeding of our hogs, and so far as known, especially in those raised in the West. Our duty is plain. Seek the cause, persist in it until found, for it can be found. Once found, prevention is easy. Keep the hogs from access to the cause. There are certain minor causes which scarcely need to be touched upon here. They may be sought in swill feeding when it contains trichinous pork; in having the family vaults enter pig pens; in the cohabitation of trichinous pigs with healthy ones, etc.

These have no relation to trichiniasis in the Western hog, but have to the Eastern. The "rat theory" is a humbug.

Still another question should be touched upon here, and that is legislation. The Washington Government cannot legislate upon this subject, much as it is necessary. It can investigate, however, and with the assistance of the State authorities, inaugurate careful examinations of the hogs in every State. There are such things as trichinous centres in the pork-raising States. These can be discovered. To these we must go "grub hunting," and that means hunting for the source from which the hog becomes infected. State legislation—but it must be universal—can meet the evil. Even the large packing-houses must come under this rule. We must have the same law for pork destined for our home as for foreign consumption. Who knows what is to be its destiny when cured? It would not be an expensive job for every packing-house to have its inspection-room. Ten girls can be accurately taught to do the work in two days, a few trusty men to collect the specimens, a duplicate check system as for railroad baggage, a branding-iron "Trichinous" for condemned hogs, and the work is done. Such hogs can be justifiably tried out for lard.

Self-interest should lead the packing-houses to inaugurate such a system, and to have it honestly carried out. It is to the advantage of the whole pork interest to look this matter squarely in the face, and not to evade the question, or seek to gain a protective legislation of the "you hit me and I'll hit you" type, against Germany or any other country. Let us be honest; let us look this evil squarely in the face, accept it, and seek by diligent research to discover the real cause of porcine trichiniasis, and thus to do away with it.

Let the United States Government do its best to have the honor of discovering the cause of trichiniasis—an American donation to the facts of science.

If we are true to ourselves, we shall be true to the whole world.

BILLINGS.

